# Effect of perioperative flurbiprofen axetil on long‐term survival of patients with esophageal carcinoma who underwent thoracoscopic esophagectomy: A retrospective study

**DOI:** 10.1002/jso.26553

**Published:** 2021-06-18

**Authors:** Yanhu Xie, Di Wang, Chen Gao, Jicheng Hu, Min Zhang, Wei Gao, Shuhua Shu, Xiaoqing Chai

**Affiliations:** ^1^ Department of Anesthesiology Anhui Provincial Hospital Hefei Anhui China; ^2^ Division of Life Sciences and Medicine, The First Affiliated Hospital of University of Science and Technology of China University of Science and Technology of China Hefei Anhui China

**Keywords:** esophageal carcinoma, flurbiprofen axetil, NSAIDs, OS, RFS, thoracoscopic surgery

## Abstract

**Background and Objectives:**

Nonsteroidal anti‐inflammatory drugs (NSAIDs) have an anti‐inflammatory response, but it remains unclear whether the perioperative use of flurbiprofen axetil can influence postoperative tumor recurrence and survival in esophageal carcinoma. We aimed to explore the effect of perioperative intravenous flurbiprofen axetil on recurrence‐free survival (RFS) and overall survival (OS) in patients with esophageal carcinoma who underwent thoracoscopic esophagectomy.

**Methods:**

This retrospective study included patients who underwent surgery for esophageal carcinoma between December 2009 and May 2015 at the Department of Thoracic Surgery, Anhui Provincial Hospital. Patients were categorized into a non‐NSAIDs group (did not receive flurbiprofen axetil), single‐dose NSAIDs group (received a single dose of flurbiprofen axetil intravenously), and multiple‐dose NSAIDs group (received multiple doses of flurbiprofen).

**Results:**

A total of 847 eligible patients were enrolled. Univariable and multivariable analyses revealed that the intraoperative use of flurbiprofen was associated with long‐term RFS (hazard ratio [HR]: 0.56, 95% confidence interval [CI]: 0.42–0.76, *p* = .001) and prolonged OS (HR: 0.49, 95% CI: 0.38–0.63, *p* = .001).

**Conclusions:**

Perioperative flurbiprofen axetil therapy may be associated with prolonged RFS and OS in patients with esophageal carcinoma undergoing thoracoscopic esophagectomy.

## 1 INTRODUCTION

Esophageal carcinoma, the fourth most common malignancy in China,^1^ is characterized by a high degree of locoregional recurrence, distant metastases, and poor overall survival (OS).^2^, ^3^ Being the sixth most common cause of cancer‐related mortalities, it accounts for an estimated 400,000 cases (4.9%) of death worldwide.^1^ Reportedly, approximately 80% of new cases occur in less‐developed regions of the world, out of which about 60% of them occur in China.^4^ Among the two dominant histologic subtypes (esophageal adenocarcinoma and esophageal squamous cell carcinoma [ESCC]),^5^ ESCC remains the main subtype with approximately 90% of cases occurring in the Asia–Pacific region, including China.^6^ China presents a high incidence of esophageal carcinoma, especially in rural regions. Furthermore, higher incidence rates of ESCC have been reported in different provinces of China, including Hebei, Henan, Fujian, and Chongqing, followed by Xinjiang, Jiangsu, Shanxi, Gansu, and Anhui,^7^ with recognized hotspots in Linxian (Henan province) and Cinxian county, and near Taihang Mountains.^8^, ^9^ The profound heterogeneity of etiological risk factors underlying esophageal carcinoma is particularly compelling. Despite its rapidly progressive course, the involvement of modifiable risk factors in conjunction with late‐stage presentation highlights the scope for better management of the disease.^10^ Several decades of extensive research in the high‐risk areas of China have provided novels insights on the epidemiology, etiology, early detection,^11^ and management of this disease.^12^, ^13^, ^14^, ^15^, ^16^, ^17^ However, despite recent advances in the diagnosis and treatment of this neoplastic condition, failure of chemotherapy and radiotherapy leads to tumor recurrence and poor prognosis. This could be mainly due to its aggressive nature and the limited efficiency of treatment modalities.^18^

Currently, esophagectomy is the mainstay of treatment for patients with resectable esophageal carcinoma, although distant control and complete resection rate continue to remain a challenge.^19^, ^20^ Nevertheless, the overall 5‐year survival rate after esophagectomy remains about 21% in China.^21^ This dismal result could be attributed to recurrence after resection of the primary tumor. Locoregional recurrences or/and distant organ metastases were found in approximately 50% of patients within 2–3 years of surgery.^22^, ^23^, ^24^ The recurrence patterns following esophagectomy have been well‐studied.^22^, ^24^, ^25^ Factors such as histologic tumor depth invasion,^22^, ^24^ local‐regional lymph node metastases,^24^ and intramural metastasis^25^ have been shown to predict tumor recurrence. Of note, recent improvements in perioperative management have reduced postoperative mortality to acceptable levels. However, the perioperative period is highly vulnerable to the development of metastases, including the accelerated growth of micrometastatic disease and increased formation of new metastatic foci.^26^ Several factors, including profound depression of antitumoral cellular immunity, may contribute to this phenomenon.^27^ In addition, during the perioperative period per se, the immune function of patients could be influenced by the anesthesia management; volatile anesthetics and opioids might worsen the immunosuppression and thereby exacerbate long‐term outcome, while local anesthetics and nonsteroidal anti‐inflammatory drugs (NSAIDs) might diminish the immunosuppression and exert beneficial effects.^28^, ^29^, ^30^ Moreover, the perioperative use of β‐adrenoceptor antagonists, NSAIDs, intravenous anesthetics, and antithrombotic agents has been linked with improved survival outcomes in patients with neoplasms.^27^, ^31^ Furthermore, NSAIDs have been shown to minimize postoperative opioid consumption and further aid the strengthening of the cell‐mediated immune competence.^32^ Collectively, these findings indicate that perioperative management is crucial as it may contribute to the long‐term outcome of patients after surgery. Still, the degree of pain after thoracic surgery is very high.^33^, ^34^, ^35^ In the absence of standardized treatment, acute pain will turn into chronic pain in more than 30% of the patients,^36^, ^37^, ^38^ affecting the patient's quality of life and cooperation. Hence, analgesia must be used, but carefully and keeping oncological safety in mind.

A previous retrospective study in 327 women who underwent mastectomy for breast cancer demonstrated that the intraoperative administration of ketorolac (NSAID) significantly reduces the risk of breast cancer relapse compared with other analgesics (sufentanil, ketamine, and clonidine).^27^ Furthermore, another recent study showed that the perioperative use of dexamethasone with/without flurbiprofen axetil is associated with longer survival in patients who underwent surgery for non‐small‐cell lung carcinoma (NSCLC).^27^ Flurbiprofen axetil, an injectable prodrug of flurbiprofen,^39^ is a nonselective cyclooxygenase inhibitor used as an NSAID.^40^, ^41^ It is widely used for postoperative pain relief.^42^ A study by Tedore et al.^43^ showed that flurbiprofen exerts its analgesic effect by inhibiting prostaglandin (PG) synthesis. Nevertheless, the effect of perioperative use of NSAIDs, especially flurbiprofen, on the long‐term survival of patients with ESCC still remains elusive. Furthermore, it is unclear whether the perioperative use of flurbiprofen has an impact on the postoperative recurrence after surgery for esophageal cancer.

Therefore, in view of the above and the association between flurbiprofen and long‐term survival is uncertain, this study aimed to investigate the effect of perioperative intravenous flurbiprofen axetil on recurrence‐free survival (RFS) and OS in Chinese patients with esophageal carcinoma who underwent thoracoscopic esophagectomy.

## 2 MATERIALS AND METHODS

### 2.1 Patient population

This retrospective study included consecutive patients with esophageal carcinoma who underwent thoracoscopic esophagectomy between December 2009 and May 2015 at the Department of Thoracic Surgery, Anhui Provincial Hospital, Hefei, China. All patients aged 50 years or older with complete clinical data were included in this study. The exclusion criteria were: (1) patients with severe impairment of pulmonary and cardiac functions during preoperative evaluation (New York Heart Association classes III/IV); (2) patients with severe postoperative complications, such as anastomotic fistula, bleeding, infection, and other life‐threatening complications; (3) patients whose postoperative histopathological results revealed benign tumors; (4) patients with incomplete or missing clinical data or whose follow‐up contact information is unavailable, or whose family members declined to participate in the follow‐up survey; (5) patients who underwent palliative surgery for secondary malignancies. This study was approved by the Biomedical Ethics Committee of Anhui Medical University and was registered in the Chinese Clinical Trial Registry (ChiCTR‐IPR‐15006482). The informed consent of patients was waived by the committee owing to the retrospective design of the study.

### 2.2 Data collection

Demographic and perioperative data for all patients with esophageal carcinoma were retrieved from the medical record database of Anhui Provincial Hospital. The collected data included age, gender, body mass index, preoperative baseline blood pressure and heart rate, American Society of Anesthesiologists' physical status, stage of the tumor, the extent of lymph node invasion and metastasis, type of tumor histology, comorbidities, history of administered NSAIDs, and perioperative data (infusion of propofol, remifentanil, and the duration of surgery).

### 2.3 Groups and NSAID administration

Since not all doctors at the authors' center use flurbiprofen for analgesia during the perioperative period, some patients did not receive flurbiprofen during the perioperative period. Therefore, the patients who received or did not receive perioperative flurbiprofen during the same period could be included. According to the number of flurbiprofen axetil, patients were categorized into three groups as follows: non‐NSAIDs (did not receive flurbiprofen axetil), single‐dose NSAIDs (patients received 100 mg of flurbiprofen axetil [50 m g/5 ml]; H20041508; Beijing Tide Pharmaceutical Co., Ltd.) intravenously before induction of anesthesia], and multiple‐dose NSAIDs (patients received a dose of 100 mg of flurbiprofen axetil intravenously before induction of anesthesia and an infusion of flurbiprofen axetil [50 mg/12 h] twice a day for 2 days on the second day after surgery). Standardized anesthetic techniques were carried out, and flurbiprofen axetil was received under general anesthesia in all patients.

### 2.4 Endpoints

The primary endpoint of this study is RFS and OS. RFS was defined as the time (in months) from the date of surgery until the first recurrence or death due to oncological cause, whichever occurred first. OS was defined as the time from the date of surgery until death due to any cause. Recurrence was defined as clinical evidence of local recurrence or metastases on radiological examination.

### 2.5 Follow‐up

The survival data of all patients were collected by telephonic interview using a structured questionnaire, including short‐term comorbidity information, such as gastrointestinal distress, cardiovascular events, and respiratory complications. Postsurgery, patients were followed tri‐monthly for the first 2 years, then twice a year for 3 years, and annually thereafter. Patients were censored if they were lost in follow‐up or remained disease‐free at the end of follow‐up.

### 2.6 Statistical analyses

Data were analyzed by using the SPSS statistical software (Version 16.0, SPSS Inc.). The Kolmogorov–Smirnov test was applied to assess the normality of continuous data. The continuous data with normal distribution or non‐normal distribution are presented as mean ± *SD* or median (range). For continuous data with normal distribution, one‐way analysis of variance was used to compare the means of groups, and the LSD test was used for further pair‐wise comparison, while for continuous data with nonnormally distributed, the Kruskal–Wallis test was performed to compare variables between different groups and determine the statistical significance. Categorical data are expressed in count (percentage), and statistical significance was determined by using the *χ* ^2^ or Fisher's exact test. Univariable Cox models were used to assess the potential impact of different baseline characteristics on the outcome. Multivariable Cox proportional hazards models were then applied after adjusting for any baseline factors and intraoperative or oncological factors related to the outcome in the univariable analysis. Factors that were possibly associated with the outcomes (set as *p* ≤ .10 in univariable analysis or were regarded as clinically important) were included in the Cox proportional hazards models for multivariable analysis to identify independent factors that were associated with RFS and OS (*p* < .05). The Kaplan–Meier analyses were used to estimate RFS and OS probabilities. The log rank test was used for the comparison of RFS and OS between the NSAID (single‐ and multiple‐dose NSAIDs) and the non‐NSAID (Group A) groups or single‐ and multiple‐dose NSAIDs group. Two‐sided *p* < .05 were considered statistically significant.

## 3 RESULTS

### 3.1 General information

**Figure 1:**
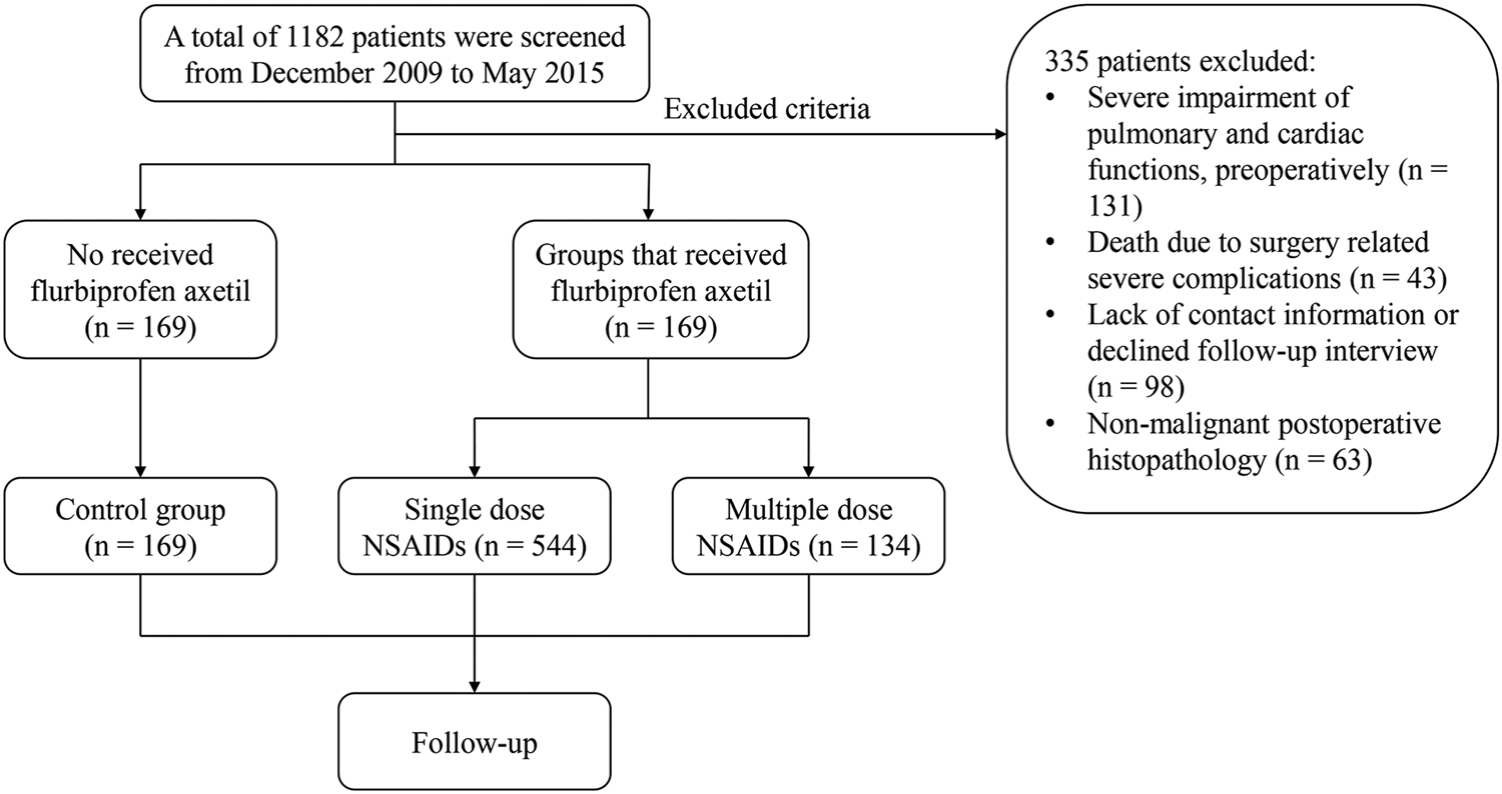
Study flow chart. NSAIDs, nonsteroidal anti‐inflammatory drugs

In this study, we retrospectively reviewed the clinical data of a total of 1182 patients. Among these, 335 patients were excluded, including 131 patients who showed severe impairment of pulmonary and cardiac functions before surgery, 43 patients died within 30 days of surgery due to severe complications (anastomotic fistula, bleeding, infection, lung embolism, acute cardio infarction), 98 patients who either declined a telephonic interview or whose contact information was unavailable, 63 patients showed nonmalignant postoperative histopathology. Finally, a total of 847 patients who met the inclusion criteria were included in our analyses (Figure 1). Among these, the non‐NSAIDs group comprised 169 patients and the groups that received flurbiprofen axetil comprised 678 patients. Out of these 678 patients, 544 patients received a single preoperative dose of flurbiprofen axetil (single‐dose NSAIDs), whereas 134 patients received multiple doses of flurbiprofen axetil (multiple‐dose NSAIDs). Table 1 summarizes baseline patient characteristics, tumor histopathological features, and perioperative data of the three groups. The mean ages of patients in the non‐NSAIDs, single‐dose NSAIDs, and multiple‐dose NSAIDs group were 62.54 ± 9.19, 63.43 ± 8.35, and 63.04 ± 8.83, respectively. Notably, significant differences with respect to esophageal cancer staging and tumor type (squamous cell carcinoma [SCC] and adenocarcinoma) were observed between the non‐NSAIDs group and the groups that received perioperative intravenous flurbiprofen axetil (single‐dose NSAIDs and multiple‐dose NSAIDs group) (*p* = .019 and *p* = .001, respectively).

**Table 1:** Characteristics of patients and perioperative procedure

Variable	Non‐NSAIDs (*n* = 169)	NSAIDs (*n* = 678)		*p* Value
		Single does NSAIDs (*n* = 544)	Multiple does NSAIDs (*n* = 134)	
Age (years)	62.54 ± 9.19	63.43 ± 8.35	63.04 ± 8.83	.490
Gender	139/30	427/117	106/28	.573
SBP (mmHg)	130.33 ± 16.86	131.07 ± 15.84	129.66 ± 11.98	.604
DBP (mmHg)	78.18 ± 9.13	78.35 ± 9.12	77.75 ± 7.88	.779
Heart rate (bpm)	76.11 ± 8.34	75.85 ± 9.28	76.04 ± 8.75	.935
BMI (kg/m^2^)	22.49 ± 2.85	22.53 ± 9.61	22.59 ± 2.97	.995
ASA grade	.063			
I	6 (4%)	9 (2%)	4 (3%)	
II	130 (77%)	443 (81%)	95 (71%)	
III	33 (19%)	92 (17%)	35 (26%)	
Tumor stage^a^	.019*			
I	4 (2)	19 (3%)	4 (3%)	
II	18 (11%)	70 (13%)	9 (7%)	
III	40 (24%)	142 (26%)	34 (25%)	
IV	97 (57%)	243 (45%)	77 (57%)	
V	10 (6%)	70 (13%)	10 (8%)	
Lymph node invasion^a^	.460			
I	108 (64%)	319 (59%)	82 (61%)	
II	41 (24%)	141 (26%)	31 (23%)	
III	18 (11%)	64 (12%)	19 (14%)	
IV	2 (1%)	20 (3%)	2 (2%)	
Metastases	.469			
Yes	2 (1%)	9 (2%)	4 (3%)	
No	167 (99%)	535 (98%)	130 (97%)	
Tumor type	.001*		
Squamous cell carcinoma	33 (20%)	420 (77%)	27 (20%)	
Adenocarcinoma (glandular cells)	136 (80%)	124 (23%)	107 (80%)	

*Note*: Continuous variables with a normal distribution (age, SBP, DBP, heart rate, BMI) are presented as mean ± standard deviation. Categorical variables are expressed as frequency or percentage.

Abbreviations: ASA, American Society of Anesthesiologists; BMI, body mass index; DBP, diastolic blood pressure; NSAIDs, nonsteroidal anti‐inflammatory drugs; SBP, systolic blood pressure.

^a^ Tumor stage and lymph node invasion classification reference from TNM‐7th Edition 2009 (UICC/AJCC) and Japanese Classification 2010 in Gastric Cancer.

* *p* < .05 was considered significant.

### 3.2 Comparison analyses of RFS and OS between controls and the groups that received flurbiprofen

**Figure 2:**
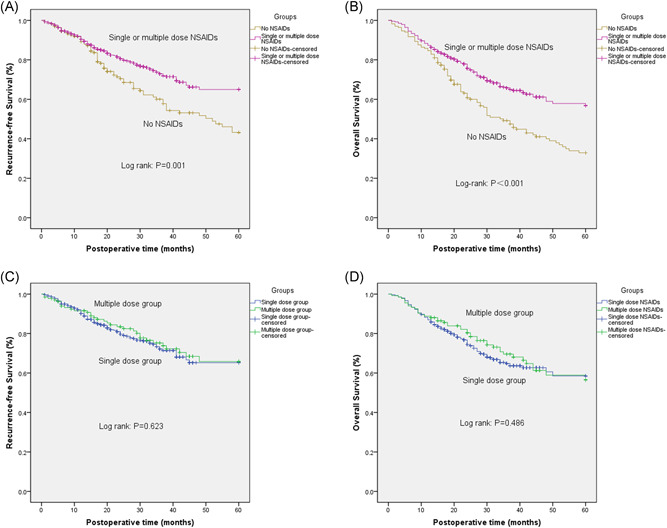
Comparison of survival curves (recurrence‐free survival [RFS] and overall survival [OS]) among the three groups. Kaplan–Meier curves for the (A) RFS and (B) OS among patients who received perioperative flurbiprofen as compared with those who did not receive the NSAID (non‐NSAIDs group). Kaplan–Meier curves for the (C) RFS and (D) OS among patients who received a single dose of perioperative flurbiprofen (single‐dose NSAIDs group) as compared with those who received multiple doses of the NSAID (multiple‐dose NSAIDs groups). NSAIDs, nonsteroidal anti‐inflammatory drugs [Color figure can be viewed at wileyonlinelibrary.com]

The intraoperative administration of flurbiprofen, either as a single dose or multiple doses, was found to be associated with long‐term RFS and OS (*p* < .001) (Figure 2A,B). Four factors that were identified by univariable analyses were included in the multivariable Cox proportional hazards model. Multivariable analysis identified three independent factors, among them increasing tumor stage (hazard ratio [HR]: 1.58, 95% confidence interval [CI]: 1.32–1.88, *p* = .001), lymph node invasion (HR: 1.51, 95% CI: 1.3–1.76, *p* = .001) were found to be significant risk factors affecting RFS in patients. Furthermore, the intraoperative administration of flurbiprofen (HR: 0.56, 95% CI: 0.42–0.76, *p* = .001) was found to be protective factors associated with long‐term RFS in these patients (Table 2).

**Table 2:** Univariate and multivariate analyses of prognostic factors associated with RFS in non‐NSAIDs and NSAIDs groups

Variable	Univariate analyses			Multivariate analysis		
	HR	95% CI	*p* Value	HR	95% CI	*p* Value
No vs. NSAIDs	0.608	0.452–0.817	.001*	0.562	0.418–0.756	.001*
Age (years)	1.004	0.988–1.020	.644	–	–	–
Gender	0.940	0.669–1.322	.723	–	–	–
SBP (mmHg)	1.003	0.994–1.012	.541	–	–	–
DBP (mmHg)	1.001	0.986–1.017	.849	–	–	–
Heart rate (bpm)	1.004	0.989–1.019	.572	–	–	–
BMI (kg/m^2^)	0.974	0.927–1.022	.279	–	–	–
ASA grade	1.065	0.773–1.466	.701	–	–	–
Tumor stage	1.684	1.424–1.992	.001*	1.577	1.322–1.882	.001*
Lymph node invasion	1.643	1.419–1.903	.001*	1.515	1.300–1.765	.001*
Metastases	1.282	0.477–3.450	.622	–	–	–
Tumor type	0.621	0.474–0.816	.001*	–	–	–
Complications	0.966	0.734–1.270	.803	–	–	–

Abbreviations: ASA, American Society of Anesthesiologists; BMI, body mass index; CI, confidence interval; DBP, diastolic blood pressure; HR, hazard ratio; NSAIDs, nonsteroidal anti‐inflammatory drugs; RFS, recurrence‐free survival; SBP, systolic blood pressure.

* *p* < .05 was considered significant.

Six factors that were identified by univariable analyses were included in the multivariable Cox proportional hazards model. Multivariable analysis identified four independent factors. Among them, increasing tumor stage (HR: 1.49, 95% CI: 1.28–1.74, *p* = .001), lymph node invasion (HR, 1.63, 95% CI: 1.43–1.85, *p* = .001), age (HR: 1.03, 95% CI: 1.01–1.05, *p* = .001) were associated with shortened OS. On the contrary, the perioperative use of flurbiprofen axetil (HR: 0.49, 95% CI: 0.38–0.63, *p* = .001) was found to be associated with prolonged OS (Table 3).

**Table 3:** Univariate and multivariate analyses of prognostic factors associated with OS in non‐NSAIDs and NSAIDs groups

Variable	Univariate analyses			Multivariate analysis		
	HR	95% CI	*p* Value	HR	95% CI	*p* Value
No vs. NSAIDs	0.571	0.445–0.733	.001*	0.492	0.382–0.634	.001*
Age (years)	1.030	1.015–1.045	.001*	1.029	1.014–1.045	.001*
Gender	0.843	0.625–1.139	.266	–	–	–
SBP (mmHg)	1.007	0.999–1.015	.069	–	–	–
DBP (mmHg)	0.996	0.983–1.009	.552	–	–	–
Heart rate (bpm)	1.005	0.992–1.018	.465	–	–	–
BMI (kg/m^2^)	0.994	0.968–1.021	.649	–	–	–
ASA grade	1.198	0.917–1.566	.185	–	–	–
Tumor stage	1.652	1.431–1.908	.001*	1.494	1.284–1.739	.001*
Lymph node invasion	1.712	1.514–1.936	.001*	1.625	1.427–1.849	.001*
Metastases	1.695	0.800–3.588	.168	–	–	–
Tumor type	0.630	0.499–0.795	.001*	–	–	–
Complications	0.764	0.606–0.964	.023*	0.812	0.636–1.037	.095

Abbreviations: ASA, American Society of Anesthesiologists; BMI, body mass index; CI, confidence interval; DBP, diastolic blood pressure; HR, hazard ratio; NSAIDs, nonsteroidal anti‐inflammatory drugs; OS, overall survival; SBP, systolic blood pressure.

* *p* < .05 was considered significant.

### 3.3 Comparison analyses of RFS and OS between single‐dose NSAID group and multiple‐dose NSAID group

Interestingly, there was no significant difference between the RFS of patients who received a single dose of flurbiprofen and that of those who received multiple doses (Figure 2C). Similarly, the frequency of flurbiprofen dosage was not found to influence the OS in these patients (Figure 2D). Three factors that were identified by univariable analyses were included in the multivariable Cox proportional hazards model. Multivariable analysis revealed increasing tumor stage (HR: 1.64, 95% CI: 1.33–2.03, *p* < .001) and lymph node invasion (HR: 1.51, 95% CI: 1.26–1.78, *p* < .001) to be significant risk factors affecting RFS in these patients (Table 4).

**Table 4:** Univariate and multivariate analyses of prognostic factors associated with RFS in single‐dose NSAIDs and multiple‐dose NSAIDs groups

Variable	Univariate analyses			Multivariate analysis		
	HR	95% CI	*p* Value	HR	95% CI	*p* Value
Single‐dose vs. multiple	0.933	0.624–1.395	.735	–	–	–
Age (years)	1.013	0.994–1.033	.186	–	–	–
Gender	0.952	0.638–1.422	.810	–	–	–
SBP (mmHg)	1.003	0.992–1.013	.644	–	–	–
DBP (mmHg)	0.995	0.977–1.013	.585	–	–	–
Heart rate (bpm)	1.005	0.987–1.022	.615	–	–	–
BMI (kg/m^2^)	0.957	0.901–1.016	.152	–	–	–
ASA grade	1.176	0.803–1.722	.406	–	–	–
Tumor stage	1.800	1.470–2.205	<.001*	1.643	1.331–2.028	<.001*
Lymph node invasion	1.697	1.433–2.009	<.001*	1.507	1.263–1.797	<.001*
Metastases	1.169	0.373–3.669	.789	–	–	–
Tumor type	0.695	0.484–1.000	.050*	–	–	–
Complications	0.978	0.707–1.353	.891	–	–	–

Abbreviations: ASA, American Society of Anesthesiologists; BMI, body mass index; DBP, diastolic blood pressure; NSAIDs, nonsteroidal anti‐inflammatory drugs; RFS, recurrence‐free survival; SBP, systolic blood pressure.

* *p* < .05 was considered significant.

All factors that were identified by univariable analyses were found to be independently associated with OS in multivariable analysis. Expectedly, increasing tumor stage (HR: 1.54, 95% CI: 1.28–1.85, *p* = .001), lymph node invasion (HR: 1.62, 95% CI: 1.40–1.89, *p* = .001), age (HR: 1.03, 95% CI: 1.01–1.05, *p* = .001) were associated with shortened OS (Table 5).

**Table 5:** Univariate and multivariate analyses of prognostic factors associated with OS in single‐dose NSAIDs and multiple‐dose NSAIDs groups

Variable	Univariate analyses			Multivariate analysis		
	HR	95% CI	*p* Value	HR	95% CI	*p* Value
Single‐dose vs. multiple	0.886	0.628–1.249	.489	–	–	–
Age (year)	1.031	1.013–1.049	.001*	1.031	1.013–1.049	0.001*
Gender	0.817	0.569–1.173	.274	–	–	–
SBP (mmHg)	1.005	0.996–1.014	.313	–	–	–
DBP (mmHg)	0.989	0.974–1.005	.170	–	–	–
Heart rate (bpm)	1.007	0.992–1.023	.375	–	–	–
BMI (kg/m^2^)	0.992	0.958–1.027	.632	–	–	–
ASA grade	1.088	0.778–1.522	.623	–	–	–
Tumor stage	1.748	1.468–2.081	<.001*	1.546	1.288–1.856	<.001*
Lymph node invasion	1.774	1.538–2.047	<.001*	1.624	1.397–1.887	<.001*
Metastases	1.823	0.809–4.111	.148	–	–	–
Tumor type	0.784	0.569–1.081	.138	–	–	–
Complications	0.787	0.595–1.042	.094	–	–	–

Abbreviations: ASA, American Society of Anesthesiologists; BMI, body mass index; DBP, diastolic blood pressure; NSAIDs, nonsteroidal anti‐inflammatory drugs; OS, overall survival; SBP, systolic blood pressure.

* *p* < .05 was considered significant.

## 4 DISCUSSION

This retrospective study demonstrated an association between the perioperative administration of flurbiprofen and a reduced risk of recurrence after surgery for esophageal carcinoma. Similarly, an apparent association was found between the intraoperative use of flurbiprofen and prolonged OS (*p* = .001). Our findings were consistent with the results of previous studies that demonstrated significant associations between the perioperative use of NSAIDs and survival outcomes (RFS and OS) of patients who underwent surgery for breast cancer and NSCLC.^27^, ^28^ On the contrary, we observed that there were no significant differences in RFS and OS of patients who received a single dose of flurbiprofen and that of those who received multiple doses. To the best of our knowledge, this is the first retrospective study to investigate and compare the effects of single and multiple doses of flurbiprofen on RFS and OS of Chinese patients who underwent thoracoscopic esophagectomy for esophageal carcinoma. Most importantly, our findings highlight the dose‐independent effect of perioperative flurbiprofen axetil therapy, which may improve patients' long‐term survival (RFS and OS) after thoracoscopic esophagectomy.

One of the most prominent prognostic factors affecting the treatment of esophageal cancer is lymph node metastasis.^44^ The presence and number of metastatic lymph nodes governing the lymph node status have been shown to be independent predictors for long‐term survival.^40^, ^45^, ^46^ Lymph node metastasis has been shown to be associated with poor survival,^47^ whereby an increasing number of metastatic lymph nodes predicted a progressively poor prognosis.^48^ Reportedly, the 5‐year survival rate for patients with lymph node metastasis is quite low. On average, patients with a single lymph node metastasis showed a significantly longer survival compared with those having two or more lymph node metastases.^49^ Similarly, a study by Zhang et al.^50^ demonstrated a significant association of the number of positive lymph nodes with survival in patients with esophageal SCC and further showed that patients with 0, 1, and ≥2 positive nodes had 5‐year survival rates of 59.8%, 33.4%, and 9.4%, respectively. Consistent with the previous literature, in this study, we demonstrated that the extent of lymph node invasion significantly affected the RFS or OS and was found to be significantly associated with shortened survival in patients who did not receive a perioperative intravenous infusion of flurbiprofen axetil. The patients' RFS and OS were improved by flurbiprofen axetil used in perioperative; however, multiple‐dose of flurbiprofen axetil cannot again extend the RFS or OS of patients who underwent surgery for esophageal carcinoma. Taken together, this study suggests that both the status of lymph nodes and the nature of a tumor need to be evaluated for optimal treatment decisions.

Staging in esophageal cancer depends on the depth of tumor invasion, involvement of regional lymph nodes, and the presence or absence of metastasis.^51^ In this study, we showed that the tumor stage could have a significant impact on both the OS and RFS of patients who did not receive a perioperative intravenous infusion of flurbiprofen axetil.

Emerging evidence suggests that the perioperative timeframe is a crucial facilitator of metastatic progression, with several deleterious processes, including excess and maladaptive perioperative responses operating at the paracrine, endocrine, and immunological levels.^52^ Several perioperative risk factors, such as shedding of malignant cells, accelerated malignant tissue proliferation and surgical stress induced due to catecholamines, PG, and opiates/opioids, excessive release of proangiogenic/proinvasive factors, increased invasion capacity, the abundant release of growth factors, psychological distress, and suppression of CMI, secretion of vascular endothelial growth factor may act synergistically to initiate new metastases and facilitate the outbreak of pre‐existing micrometastases, thus impacting the long‐term recurrence rates in patients with esophageal carcinoma.^53^

Substantive clinical and epidemiological evidence indicates that the increase in the levels of proinflammatory mediators, such as cytokines, chemokines, and PGs in solid tumors, has become a major risk factor for cancer development.^54^ A review of the literature reveals that PGs, especially prostaglandin E2 (PGE_2_), produced by COX‐2 (a member of the cyclooxygenase enzyme family), promote neoplastic progression.^55^ The role of COX‐2 in carcinogenesis, especially in cell proliferation, apoptosis inhibition, angiogenesis, invasiveness, and immunosuppression, has been well‐studied.^56^ Furthermore, the overexpression of COX‐2 was found to be significantly associated with the depth of invasion, lymph node metastasis, distant metastasis, and TNM stage in esophageal cancer.^57^ Moreover, a number of the study suggested that COX‐2 overexpression was associated with a poor prognosis.^58^, ^59^, ^60^ A recent study indicated that perioperatively administered flurbiprofen inhibited COX‐2 and PGE_2_ levels that in turn impaired the postoperative increase of programmed death 1 (PD‐1) expression levels on circulating CD8^+^ T cells.^61^ Further, another study showed that circulating T cells (CD4^+^T and CD8^+^T cell) express upregulated levels of PD‐1, which correlated with a poorer clinical outcome in patients with lung cancer.^62^ Of note, a large proportion of patients (14.5%–82.8%) with esophageal carcinoma harbor tumors with programmed death‐ligand 1 (PD‐L1) expression.^63^ A study by Hsieh et al.^63^ advocated that the overexpression of PD‐L1 on cytoplasm is an independent prognostic factor for disease‐free survival in patients who underwent esophagectomy for ESCC and further suggested that patients without overexpression of PD‐L1 and PD‐L2 had a better OS. PD‐1 expression in tumor‐infiltrating T cells and circulating T cells has been reported in several malignant tumors.^64^ In addition, the expression of PD‐L1 (ligand for PD‐1) has been linked with rapid cancer progression, higher recurrence rates, and worse survival.^63^ Corroborating the results of this study, these findings collectively indicate that perioperative flurbiprofen therapy may modify the immune‐checkpoint expression and limit long‐term cancer recurrence, thus improving the OS rates in patients undergoing esophagectomy for esophageal carcinoma.

Expectedly, in this study, multivariable analyses showed age to be independently associated with poorer survival in patients who did not receive perioperative flurbiprofen. After adjustment for the potential confounder (age), the association between perioperative flurbiprofen administration and the lower risk of cancer recurrence and prolonged survival remained significant. Advanced age is associated with poor outcomes following esophageal resection.^65^ Moreover, advanced age is considered a relative contraindication for flurbiprofen (and other NSAIDs) and consequently prompts a decreased usage of flurbiprofen in elderly patients. Therefore, the perioperative use of flurbiprofen in elderly patients with esophageal cancer after surgical resection can improve survival, but dose control and potential risk monitoring are essential.

In addition to the significant findings revealed by this study, there are some limitations currently study. Despite accessing high‐quality electronic health record databases and considering all‐known variables that could affect the outcome in our statistical analyses, a series of potential uncontrolled and unrecognized biases, such as selection bias, diagnostic bias, and bias in follow‐up, which may confound the results were inevitable due to the inherent nature of this observational retrospective study. Furthermore, the single‐center study design may limit the generalizability of the findings in this study. Moreover, the follow‐up data regarding patients' neoadjuvant chemotherapy, adjuvant radiation, or other adjuvant therapies were unavailable, which might influence the study's endpoint. Third, study groups received either a single equivalent dose of intravenous flurbiprofen preoperatively or multiple doses of flurbiprofen with 100 mg both preoperatively and postoperatively (twice a day for 2 days). As the treatment was uniform, concerning the individual difference, individualized dosage regimens based on the patient's status and the tumor nature need to be considered to minimize the potential bias.

## 5 CONCLUSIONS

In summary, this study demonstrated significant associations between the intraoperative administration of flurbiprofen and RFS and OS of Chinese patients who underwent thoracoscopic esophagectomy for esophageal carcinoma. The findings reported here showed new light on the effect of perioperative flurbiprofen axetil therapy, which may improve patients' long‐term survival after thoracoscopic esophagectomy. Further prospective randomized controlled trials for ascertaining the efficacy of perioperative flurbiprofen axetil, validating it as a useful adjuvant in the immune‐checkpoint blockade therapy and further establishing it as a standard‐of‐care treatment in patients undergoing thoracoscopic esophagectomy are necessary.

## CONFLICT OF INTERESTS

The authors declare that there are no conflict of interests.

## AUTHOR CONTRIBUTIONS

Xiaoqing Chai and Yanhu Xie were responsible for the study concept and design. Wei Gao, Yanhu Xie contributed to data collection. Di Wang and Jicheng Hu conducted the statistical analyses. Jicheng Hu and Di Wang drafted the manuscript and contributed to the interpretation of the results. Xiaoqing Chai reviewed this manuscript. All authors approved the final manuscript.

## SYNOPSIS

To explore the effect of perioperative intravenous flurbiprofen axetil on recurrence‐free survival (RFS) and overall survival (OS) in patients with esophageal carcinoma. Univariable and multivariable analyses revealed that the intraoperative use of flurbiprofen was associated with long‐term RFS and prolonged OS. There were no significant differences in RFS and OS of patients who received a single dose of flurbiprofen and that of those who received multiple doses.

## Data Availability

The data that support the findings of this study are available on request from the corresponding author.
